# Role of the long cytoplasmic domain of the SIV Env glycoprotein in early and late stages of infection

**DOI:** 10.1186/1742-4690-4-94

**Published:** 2007-12-14

**Authors:** Andrei N Vzorov, Armin Weidmann, Natalia L Kozyr, Vladimir Khaoustov, Boris Yoffe, Richard W Compans

**Affiliations:** 1 Dept. of Microbiology and Immunology and Emory Vaccine Center, Emory University, Atlanta, GA, USA; 2 Dept of Medicine and Emory Vaccine Center, Emory University, Atlanta, GA, USA; 3 Dept of Medicine, Baylor College of Medicine, Houston, TX, USA; 4 MorphoSys AG, Martinsried/Planegg, Germany

## Abstract

**Background:**

The Env glycoproteins of retroviruses play an important role in the initial steps of infection involving the binding to cell surface receptors and entry by membrane fusion. The Env glycoprotein also plays an important role in viral assembly at a late step of infection. Although the Env glycoprotein interacts with viral matrix proteins and cellular proteins associated with lipid rafts, its possible role during the early replication events remains unclear. Truncation of the cytoplasmic tail (CT) of the Env glycoprotein is acquired by SIV in the course of adaptation to human cells, and is known to be a determinant of SIV pathogenicity.

**Results:**

We compared SIV viruses with full length or truncated (T) Env glycoproteins to analyze possible differences in entry and post-entry events, and assembly of virions. We observed that early steps in replication of SIV with full length or T Env were similar in dividing and non-dividing cells. However, the proviral DNA of the pathogenic virus clone SIVmac239 with full length Env was imported to the nucleus about 20-fold more efficiently than proviral DNA of SIVmac239T with T Env, and 100-fold more efficiently than an SIVmac18T variant with a single mutation A239T in the SU subunit and with a truncated cytoplasmic tail (CT). In contrast, proviral DNA of SIVmac18 with a full length CT and with a single mutation A239T in the SU subunit was imported to the nucleus about 50-fold more efficiently than SIVmac18T. SIV particles with full length Env were released from rhesus monkey PBMC, whereas a restriction of release of virus particles was observed from human 293T, CEMx174, HUT78 or macrophages. In contrast, SIV with T Envs were able to overcome the inhibition of release in human HUT78, CEMx174, 293T or growth-arrested CEMx174 cells and macrophages resulting in production of infectious particles. We found that the long CT of the Env glycoprotein was required for association of Env with lipid rafts. An Env mutant C787S which eliminated palmitoylation did not abolish Env incorporation into lipid rafts, but prevented virus assembly.

**Conclusion:**

The results indicate that the long cytoplasmic tail of the SIV Env glycoprotein may govern post-entry replication events and plays a role in the assembly process.

## Background

The Env glycoproteins of retroviruses play an important role in the initial steps of infection involving the binding to cell surface receptors and entry by membrane fusion. The Env glycoprotein also plays an important role in viral assembly at a late step of infection. There is evidence for intracellular interaction of Env with the matrix protein [1-4], and the Env glycoprotein directly influences the site of release of virus particles in polarized epithelial cells [5]. The cytoplasmic tail of the Env glycoprotein is required for such interactions and has effects on Env incorporation and infectivity [3,6]. In addition, removal of the cytoplasmic domain can increase the expression of Env on the surface of infected cells, its incorporation into VLPs or membrane vesicles [7-9] and the fusion activity of the Env glycoprotein [10,11].

SIV and HIV Env glycoproteins contain a relatively long cytoplasmic domain (150–200 amino acids) compared with most other retroviral Env glycoproteins. Nonhuman primates in Africa that are natural hosts for SIV appear to be disease resistant when infected with SIV, whereas nonnatural Asian macaque hosts such as rhesus macaques exhibit progressive CD4^+^-T-cell depletion and AIDS [12-14]. When SIV strains were passaged on human cell lines they frequently acquired a premature stop codon and expressed a truncated Env glycoprotein that lacks all but approximately 20 amino acids of the cytoplasmic domain [15-18]. However, molecular clones of SIV with truncated Env only establish transient infection in rhesus macaques [19]. Variants with truncated Env are commonly isolated from both types of infected monkeys [15,17,19]. However, variants of HIV with truncated Env are rarely isolated from infected patients, even though HIV-1 infected patients can harbor viruses with truncated Env that are able to mediate CD4-independent infection of CD8^+ ^cells [20].

By budding through lipid rafts in T-cells, HIV and SIV selectively incorporate raft marker proteins and exclude non-raft proteins [21]. The depletion of cholesterol from viral membranes inactivates and permeabilizes HIV and SIV virions [22]. These results indicate a critical role of lipid rafts in the biology of these viruses. It was reported that HIV budding in primary macrophages occurs through the exosome release pathway [23]. A non-pathogenic molecular clone SIVmac1A11 closely related to SIVmac239 but with a truncated Env, which was isolated from an infected rhesus macaque, was able to replicate in monkey macrophages, rhesus PBMC, and human T-cells. However, a pathogenic clone of SIVmac239 was restricted for replication in monkey macrophages and human T-cells [16,17,24]. These results indicated that virus replication capacity in different cell lines does not correlate with *in vivo *virulence.

In the present study we have compared molecularly cloned SIV isolates with sequence differences in the Env glycoprotein, acquired during adaption to human T cells, to investigate the effects of the long cytoplasmic tail of the Env glycoprotein on early steps of replication as well as assembly of SIV. We further compared the replication of these viruses in dividing and non-dividing cells.

## Results

### Properties of SIV variants

**Figure 1:**
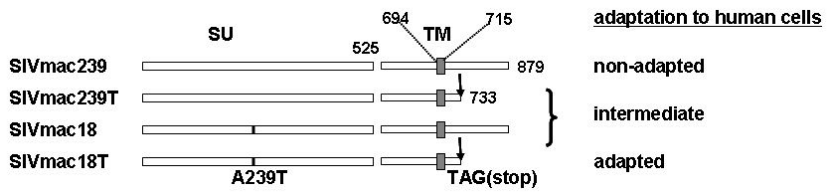
**Schematic representation of envelope gene products of cloned SIV adapted or not adapted to human cells**. SIVmac239 has a full length 164 amino acid cytoplasmic tail (CT) [64]. The 239T construct has a truncated CT of 18 amino acids. A site-specific C to T mutation present in the 239T *env *gene changed a CAG glutamine codon at position 734 to a TAG termination codon. SIVmac18T contains a single amino acid substitution A239T in the SU domain designated 18 [25]. Numbers represent amino acid residues. Shaded boxes represent the hydrophobic transmembrane-spanning regions.

In the present study we compared SIVmac239 and several SIVmac239 derivates with mutations in the Env glycoprotein resulting from adaptation to cell culture (Fig. 1). SIVmac18 with a single mutation A239T in the SU subunit and a full length cytoplasmic tail, SIVmac18T with a single mutation A239T in the SU subunit and with a truncated cytoplasmic tail, and SIVmac239T with a truncated cytoplasmic tail were described previously [25]. SIVmac239 exhibits a low level of Env incorporation, resistance to neutralization by antibodies and slow replication in human CEMx174 and rhesus monkey PBMC (Table 1). SIVmac18T, a variant with a truncated Env isolated by adaptation to human HUT78 cells, exhibits a high level of Env incorporation, sensitivity to neutralization and rapid replication in human HUT78, CEMx174 and rhesus monkey PBMC. SIVmac18, the corresponding virus with a full length Env, also demonstrated a high level of Env incorporation and sensitivity to neutralization, but slow replication.

**Table 1:** Phenotypic properties of SIV.

*Virus*	*Phenotypic properties*^1^			
	Env incorporation	length of Env CT	sensitivity to neutralization	replication^2^
SIVmac239	low	full	low	slow
SIVmac239T	high	truncated	low	slow
SIVmac18	high	full	high	slow
SIVmac18T	high	truncated	high	rapid

^1^properties of SIV variants were determined previously (Vzorov et al., 2005)

^2^levels of replication were determined in HUT78, CEMx174 cells, and rhesus monkey PBMC

### SIV post-entry replication in dividing vs. non-dividing cells

**Figure 2:**
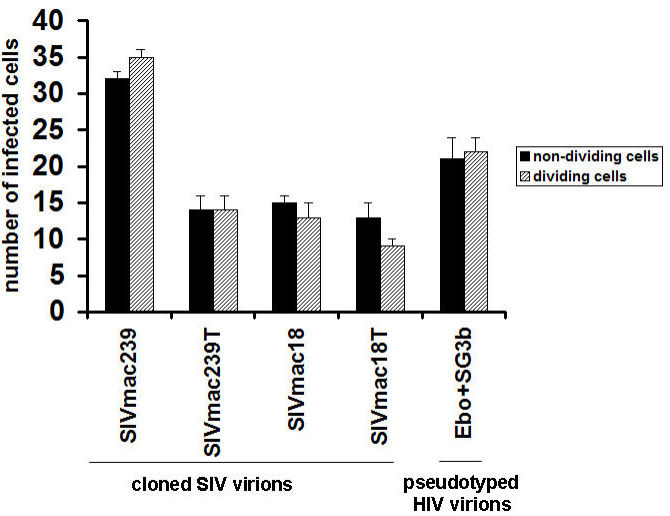
**Infectivity of SIV with full length or truncated Env and pseudotyped HIV virions in dividing and non-dividing cells**. MAGI-R5 cells treated or untreated with aphidicolin were infected with SIVs or pseudotyped HIV virions. For inoculation of cells, each virus was used at a similar titer determined as described in Methods. Infectivity of SIV and HIV was measured by removal of the media after three days, fixation and staining of cells with X-gal [29]. The infectivity was determined by counting the number of infected cells in wells inoculated with viruses. Data are plotted as the mean of three experiments, each replicated twice. Error bars represent standard deviations.

**Figure 3:**
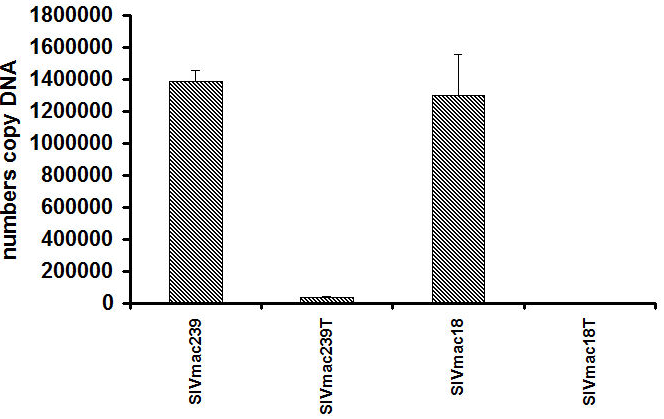
**Comparison of early steps of replication of SIV with full length or truncated Env in rhesus monkey PBMC**. Rhesus monkey PBMC (3 × 10^6^) were inoculated by SIV with full length or truncated Env with an equal infectious index (IU/ng) using ~3 IU/ng for each virus as described in Methods. Samples of nuclear DNA were tested for the presence of SIV DNA by real-time PCR in a TaqMan thermal cycler at 24 h after infection. Nuclear DNA samples corresponding to equal numbers of cells infected by SIV were analyzed in triplicate. Fluorescence was recorded as a function of PCR amplification cycle. Quantitative SIV determinations were made by comparison with a standard curve produced by using serial dilution of plasmid DNA.

**Figure 4:**
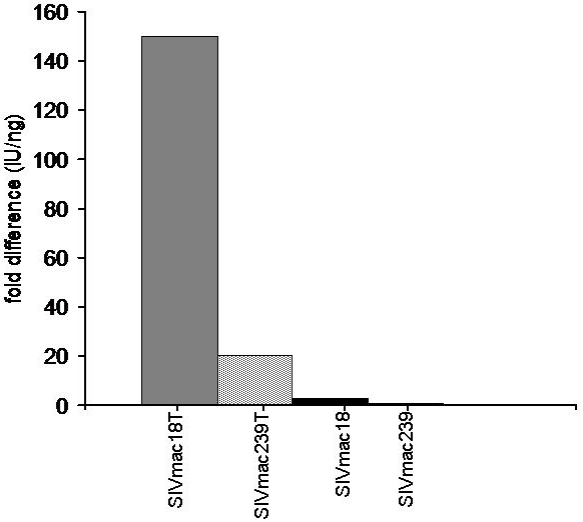
**Analysis of efficiency of SIV replication in dividing vs non-dividing CEMx174 cells**. CEMx174 cells (2 × 10^6^) treated or untreated with aphidicolin were inoculated by SIV with full length or truncated Env with similar titer; the amounts of input virus was determined based on the infectious index (IU/ng) as described in Methods. At 24 h after infection samples of nuclear DNA were tested for the presence of SIV DNA by real-time PCR in a TaqMan thermal cycler. Nuclear DNA samples corresponding to equal numbers of cells infected by SIV were analyzed in parallel. Fluorescence was recorded as a function of PCR amplification cycle. Quantitative SIV determinations were made by comparison with a standard curve produced by using serial dilution of plasmid DNA. The ratios of replication levels in dividing:non-dividing cells are shown.

The entry mechanisms appear to be similar for T and M tropic SIV viruses [26]. They utilize similar receptors and coreceptors for membrane fusion and are able to use the endocytic pathway [27]. The early events of SIV infection include the attachment, entry, uncoating and transport of the genome to the transcription site, formation of the preintegration complex (PIC), and import into the nucleus. Not much is known about the composition of reverse transcription complexes, particularly during the early steps after internalization. After virus-cell fusion, viral RNA and associated proteins are released into the cytoplasm and may interact with the cytoskeleton [28]. To investigate the possible effect of Env glycoprotein differences on early steps of replication in dividing and non-dividing cells we used an indicator cell line assay with human epithelial HeLa cells expressing CCR5 and CD4. The nuclear activation of a galactosidase indicator assay does not require late events such as virion protein expression, virus particle assembly, or virion maturation [29]. To compare infection in dividing or non-dividing MAGI-R5 cells, we used SIV viruses and Ebola GP pseudotyped HIV at a similar titer determined as described in Methods, to infect about 30 to 50 dividing cells. Non-dividing cells were arrested in the G_1_-S phase of the cell cycle by using aphidicolin, an inhibitor of eukaryotic DNA polymerase α. [30]. After 3 days of infection the numbers of infected cells were compared in dividing and non-dividing cells (Fig. 2). Similar levels of blue staining nuclei were observed in dividing and non-dividing cells in all samples, including cells infected by Ebola GP pseudotyped HIV. The results indicate that import of proviral DNA of SIV and HIV to the nucleus in dividing and non-dividing cells occurs by mechanisms that are independent of the differences in sequence of Env. As an alternative method, we also used real-time PCR, which is a more accurate method for comparison of early steps in replication (during 24 h post transfection) of viruses with different replication rates. We used the same amounts of input virus with an equal infectious index (IU/ng) ~3 IU/ng for each virus as described in Methods. A high number of copies of proviral DNA was determined in nuclei isolated from rhesus monkey PBMC infected by SIV with full length Env, and a significantly lower amount in nuclei infected by SIV with truncated Env at 24 hr post infection: about 1.39 × 10^6 ^DNA copies infected by SIVmac239 and about 1.3 × 10^6 ^DNA copies infected by SIVmac18, or about 4 × 10^4 ^DNA copies infected by SIVmac239T and about 5.3 × 10^3 ^DNA copies infected by SIVmac18T (Fig. 3). We obtained similar results with other tested cell lines CEMx174, HUT78, rhesus monkey macrophages (not shown); with increased multiplicity of infection for SIV viruses with truncated Env we observed increased replication levels. The ratio of infectious indices was 3 IU/ng of SIVmac239 to 9 IU/ng of SIVmac18 to 60 IU/ng of SIVmac239T to 450 IU/ng of SIVmac18T, or differences of 3 to 20 or 150 fold, respectively. We determined about 2 × 10^5 ^copy numbers per 1 × 10^6 ^dividing or non-dividing CEMx174 cells for all viruses after PCR amplification (Fig. 4). The amount of proviral DNA in nuclei isolated from dividing and non-dividing cells infected by SIV with full length or truncated Env was quite similar, within one PCR cycle. The results may also indicate the possible difference between DNA metabolism of SIV with full length or truncated Env by significantly higher ratio of infectious particles to proviral DNA copies of SIV with full length than with truncated Env.

Taken together, the results indicate that virus entry into cells was similar for SIV with full length or truncated Env in dividing vs. non-dividing cells. The full length Env glycoprotein exhibited a significant effect on the efficiency of SIV postentry replication events compared with truncated Env, but virus with truncated Env can overcome this restriction by high multiplicity of infection.

### Production of progeny SIV in dividing and non-dividing cells

To evaluate possible differences in viral particle production in dividing vs. non-dividing cells we compared the release of Gag antigen (p27) in SIV infected CEMx174 cells that were untreated or treated with aphidicolin for 24 hr before and during infection. To control for possible effects of cell viability on Gag production, a parallel MTT assay was performed. The total production of Gag was about 2-fold lower in non-dividing cells than in dividing cells infected by with full length Env SIVmac239 or with the same level in both type of cells infected by mutant SIVmac18 with full length Env (Table 2). The total production of Gag was about 2-fold higher in non-dividing cells than in dividing cells infected by SIV with truncated Env (SIVmac239T, SIVmac18T). Infection with all viruses had similar effects on viability of dividing or non-dividing (aphidicolin treated) cells; viability of cells treated with aphidicolin for 3 days was about 3-fold lower compared with cells treated for 1 day. The results indicate that release of Gag antigen into media of non-dividing cells infected by SIV with full length Env was restricted but there was no such inhibition for SIV with truncated Env.

**Table 2:** Production of Gag antigen SIV in dividing and non-dividing CEMx174 cells.

*Virus*	*MTT*^1 ^*+aphid1day/+aphid 3 days (OD)*	Viability index *(fold difference)*	*p27 ng/ml*^2 ^*-aphid.3 days*	*p27 ng/ml*^2 ^*+aphid.3 days *(x3)^3^
SIVmac239	0.327/0.106	3	28	17
SIVmac239T	0.302/0.105	2.9	32	41
SIVmac18	0.334/0.104	3.2	10	12
SIVmac18T	0.386/0.117	3.7	19	41

^1^MTT assay is described in Methods

^2^Amount of Gag p27 antigen in supernatants determined by ELISA described in Methods

^3^amount was adjusted in according to results of MTT assay.

In addition we compared Gag antigen production in monkey or human monocyte-derived macrophages infected with SIV full length or truncated Env. As a control, monkey M-tropic SIVmac1A11, a closely related strain to SIVmac239, with truncated Env and with other differences in sequence, important for macrophage-tropism was used [31]. Cell-free supernatants were harvested from the cultures at 7 days post-infection and tested for the presence of Gag p27 antigen. We observed release of Gag antigen from monkey macrophages infected by SIVmac1A11 but not from cells infected by SIVmac239, SIVmac239T, SIVmac18 or SIVmac18T (Table 3). A high level of Gag antigen was released into media of human macrophages infected by mutant SIVmac18T with truncated Env, a trace amount from cells infected by mutant SIVmac18 with full length Env, and release was not found in supernatant of cells infected by SIVmac239, SIVmac239T, or SIVmac1A11. The results indicate that SIV with truncated Env predominantly produced Gag antigen in macrophages.

**Table 3:** Production of Gag antigen SIV in macrophages.

*Virus*	*Macaque macrophages p27 ng/ml*^1^	*Human macrophages p27 ng/ml*^1^
SIV239	0	0
SIV239T	0	0
SIV18	0	2
SIV18T	0	40
SIV1A11	15	0

^1^Amount of Gag p27 antigen in supernatants (24-well plate) determined by ELISA described in Methods.

**Figure 5:**
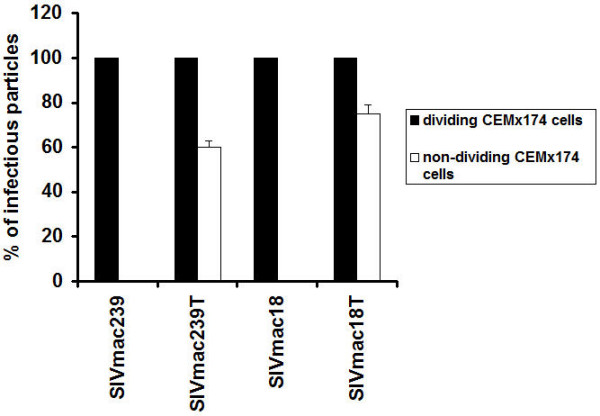
**Production of SIV infectious particles in dividing and non-dividing CEMx174 cells**. CEMx174 cells in a 96-well plate about 3 × 10^4 ^per well treated or untreated with aphidicolin were infected by SIV variants with the same titer determined as described in Methods. The supernatants were collected after 1 and 3 days post infection and the p27 content was determined by ELISA Core Antigen assay (Table 2). SIV particles with about 0.5 ng/well of p27 antigen were used for inoculation of JC-53B cells. The infectivity of particles was measured by removal of the media after 3 days, fixation and staining of cells with X-gal. The percent of particle infectivity was determined by dividing the number of infected cells in wells inoculated with particles collected from supernatants of SIV infected non-dividing cells by the number in wells inoculated with particles collected from supernatants of SIV infected dividing cells after 3 days (the maximum amount for each virus). Data are plotted as the mean of three experiments, each replicated twice.

To investigate the infectivity of particles released in the supernatant of SIV infected CEMx174 cells during 3 days of infection from the experiment described above (Table 2), we performed a replication assay in HeLa cells expressing high levels of CCR5 and CD4 (JC-53B cells). The highest number of infectious particles was produced after 3 days post infection in all SIV infected dividing cells. We observed infectious particles in the supernatant of SIVmac239 infected dividing cells only after 3 days designated (100%), and no infectious particles (0%) in the supernatant of SIVmac239 infected non-dividing cells after 1 or 3 days. The absence of infectious particles was also observed with the SIVmac18 mutant that carried a full length Env. In contrast, viruses with truncated Env (SIVmac239T, SIVmac18T) produced infectious particles starting at early times post infection, 1 or 3 days post infection in dividing as well as non-dividing cells (not shown). We observed levels of about 60% infectious particles in the supernatant of SIVmac239T and about 75% in the supernatant of SIVmac18T infected non-dividing cells after 3 days (Fig. 5). The results demonstrate that only SIV with truncated Env produced infectious particles in non-dividing CEMx174 cells, although SIV with a full length Env was able to produce and release non-infectious Gag particles in these cells.

We also compared production of infectious particles containing SIVmac239, SIVmac239T, and SIVmac18T Env in 293T epithelial cells. The virus stocks were prepared by transfection of 293T cells with similar amounts of DNA. The level of extracellular Gag in cells infected by SIVmac239 was about 3-fold higher than in cells infected by SIV239T or SIVmac18, and about 5-fold higher than in cells infected by SIVmac18T (Table 4). The infectivity titer in supernatants from transfected cells was analyzed using indicator cell lines. We found that the infectivity titer of SIV with truncated Env was about 6 to 30-fold higher than SIV with full length Env. SIV with a full length Env apparently produces reduced levels of infectious particles in human 293T cells, although total particle release was higher than in cells infected by SIV with truncated Env. Taken together, the results indicated that production of particles by SIV with full length Env was cell type dependent: particles were produced in monkey PBMC and release of particles was inhibited in human T cells and macrophages. In contrast, SIV with truncated Env produced infectious particles in all types of cells tested.

**Table 4:** Replication of SIV variants generated in human 293T cells.

*Virus^a^*	*JC-53B titer^b ^IU^c^/ml*	*ELISA (p27)^b ^ng/ml*	*IU/ng^c^*	*(fold difference from SIVmac239)*
SIVmac239	1 × 10^3^	306	3	-
SIVmac239T	6 × 10^3^	101	59	20
SIVmac18	1 × 10^3^	117	9	3
SIVmac18T	3 × 10^4^	65	461	154

^a^Viruses were obtained simultaneously after transfection of 293T cells with equimolar amounts of DNA.

^b^Assays are described in Methods.

^c^Infectious units

### Effects of modifications in the long cytoplasmic tail on lipid raft association and assembly of SIV in 293T cells

**Figure 6:**
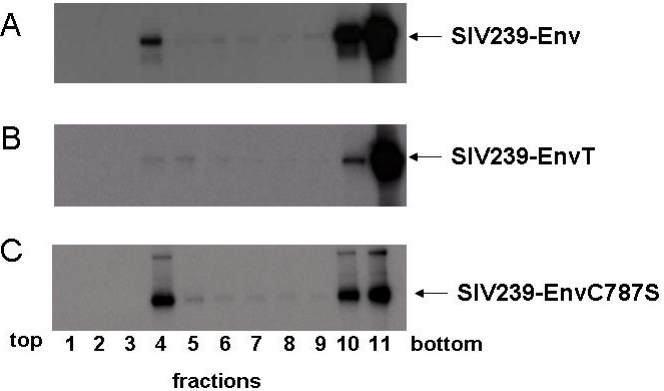
**Lipid raft association of the SIV Env protein**. The interaction of the Env protein of SIVmac239 (A), SIVmac239-EnvT (B), and SIVmac239-EnvC787S (C) with lipid rafts was analyzed in a discontinuous sucrose gradient. CEMx174 (A, B, C) cells were infected with 2 pfu/cell of respective vaccinia recombinant viruses. The infected cells were labeled with ^35^S-methionine/cysteine, disrupted by detergent TX-100 and a discontinuous sucrose gradient of 5 to 30% sucrose was used to obtain 11 fractions as described in Methods.

The SIV Env glycoprotein with a long but not with a truncated CT is palmitoylated at a single cysteine at residue position 787, which may be important for its interactions with cellular proteins. However, mutations that change the full length Env glycoprotein palmitoylation state did not alter its transport, surface expression or cell fusion activity [32]. Since palmitoylation could be involved in lipid raft association, the association of the Env glycoprotein with detergent resistant microdomains was compared for SIVmac239 with a long cytoplasmic tail (SIVmac239-Env), the Env mutant with a truncated TM glycoprotein (SIVmac239-EnvT) and a palmitoylation site mutant in which the cysteine at position 787 was changed to serine (SIVmac239-EnvC787S). These glycoproteins were found to be expressed and efficiently processed in human CEMx174 cells at similar levels (not shown). However, differences were observed in targeting of these viral envelope glycoproteins to detergent-resistant membrane microdomains (Fig. 6). The full-length wild-type as well as the palmitoylation-deficient mutant SIVmac239Env C787S glycoproteins were both found in the low-density sucrose gradient fraction, while the Env glycoprotein with a truncated cytoplasmic tail was not apparently targeted to lipid rafts, since it was not found in the low-density fractions. These results indicate that the long cytoplasmic tail of the Env glycoprotein but not its palmitoylation is required for incorporation of Env into lipid rafts. The SIV viruses with truncated Env glycoproteins are therefore able to replicate efficiently in cell lines despite their lack of Env lipid raft association.

To compare the assembly of different Env glycoproteins into virions, we transfected human 293T cells with equal amounts of proviral DNA. At 3 days post transfection cells and supernatants were collected and analyzed by RT assay (not shown). We found similar levels of RT activity in supernatants from cells infected by SIV with full length or truncated Env glycoproteins. The lowest RT activity, about 100-fold lower than in other SIV samples, was observed in supernatants from cells infected by SIV with the C787S Env mutant which eliminated palmitoylation. The infectivity titer of SIV with truncated Env was about 6 to 30-fold higher than SIV with full length Env as described above (Table 4). These results indicate that palmitoylation enhances virus replication and/or assembly viruses with full length Env but is not required in viruses with truncated Env.

### Effects of full length and truncated Env on host-cell gene expression

We also analysed the effect of Env glycoprotein differences on cellular transcriptional responses to infection. PBMC cells were infected with SIVmac239 variants with full length or truncated Env glycoproteins. Both viruses infected about 30% of cells at 6 days post infection as detected by flow cytometry. We examined mRNAs from SIVmac239 and SIVmac239T infected cells, and compared transcriptional responses to those observed in uninfected PBMC. The results were verified by real-time PCR with the same RNA samples (Table 5). The real-time PCR data confirmed that SIV with full or truncated Env induced similar cellular transcriptional responses. No changes were observed in levels of mRNA induction by SIV with full and truncated Env. These results show that the differences in the Env cytoplasmic tail did not result in major differences in effects on host-cell transcriptional responses.

**Table 5:** Comparison of mRNA responses by real-time PCR^1^.

Genes	SIVmac239T/SIVmac239 (fold difference)^2^
IL2	1.34
IL4	1.76
IL6	-1.32
IL7	-1.41
IL10	-1.05
IL12p40	1.09
IL15	-1.16
IFNA1	1.13
IFNα	1.15
IFNβ	1.04
IFNγ	-1.14
Mx	-2.22
TNFa	-1.11
IRF1	1.09
IRF2	1.05
IRF3	-1.09
IRF4	1.09
IRF5	1.13
IRF7	-1.27
PU.I	1.06
SPIB	-1.05

^1^RNA samples were the same as used for microarray assay.

^2^Changes in cellular mRNA levels after infection by SIV with full length Env were compared with mRNA levels in cells infected by SIV with truncated Env and expressed in folds.

Note fold repression is indicated by a minus sign

## Discussion

The differences in properties between SIV with full length or truncated Env have been previously studied with respect to pathogenicity [17], fusion activity [10,11], and assembly [4,9,25]. In the present study we had several goals: to study the possible role of the long cytoplasmic tail of the Env glycoprotein in post-entry events, to examine the lipid raft association of Env glycoproteins with full length or truncated cytoplasmic tails, and to compare assembly and release of SIV with full length and truncated Env in dividing and non-dividing cells. We also compared several cloned SIV viruses with sequence differences in the SU and CT subunits of the Env glycoprotein, that were related to adaptation to HUT78 cells [25].

The early steps of HIV and SIV infection include the attachment of viruses to host cells, entry and transport of the genome to the transcription site, formation of the PIC, and import to the nucleus. Electron microscopic studies showed that HIV cores were disrupted shortly after virus-cell fusion [33] and viral RNA and associated proteins were released into the cytoplasm and were likely to interact with the cytoskeleton [28]. We found that early steps in replication of SIV with full length or truncated Env were similar in dividing and non-dividing cells. Our results also indicated that internalization of SIV was correlated with amount of p24 input, but not with differences in Env glycoproteins (not shown). Previous studies also indicated that viruses might be internalized into cells irrespectively of CD4 surface expression and with almost equal efficiencies in cells susceptible or not susceptible to HIV infection [34]. The most striking differences were observed when we compared post-entry relocation of SIV with full length or truncated Env using similar input virus levels. The proviral DNA of SIVmac239 with full length Env was transported to the nucleus about 20-fold more efficiently than SIVmac239T with truncated Env, and 100-fold more efficiently than the SIVmac18T variant with a truncated cytoplasmic tail and with a single mutation A239T in the SU subunit. In contrast, the proviral DNA of SIVmac18 with a full length Env and with a single mutation A239T in the SU subunit was transported to nucleus almost as efficiently as the parental SIVmac239. Env glycoproteins are not involved in nuclear import of the HIV pre-integration complex [35], which may suggest that the effects of Env glycoproteins during early steps of SIV infection is associated with other steps in post-entry replication.

We observed release of infectious SIV particles with full length Env in monkey PBMC cells, but a restriction of particle release in human CEMx174, HUT78, epithelial 293T, or in macrophages. These results are consistent with previous studies indicating that replication of T-tropic SIV and HIV with full length Env is inhibited at a post-nuclear step in macrophages [36,37]. Our results also demonstrated that a mutation in the long cytoplasmic tail that eliminates palmitoylation did not abolish Env incorporation into lipid rafts as was described for HIV-1 [38], but prevented virus assembly. In contrast to HIV-1 [39] our results indicate that palmitoylation of the SIV Env cytoplasmic tail is not a prerequisite association with detergent insoluble microdomains. Similar results have been reported for EBV; the interaction of LMP-1 with lipid rafts was shown to be independent of palmitoylation [40]. Furthermore, palmitoylation of viral transmembrane proteins does not necessarily trigger interaction with lipid rafts, since palmitoylated VSV G protein is found in a TX-100 soluble membrane fraction [41]. Palmitoylation was critical for infectivity of SIV with full length Env, and also may impact HIV-1 infectivity [39,42]. Inhibitory factors such as TRIM5α target the CA and/p2 components of the incoming virus and presumably would be able to restrict infection of both viruses with full length and truncated Env [43,44].

In contrast to SIV with full length Env, similar levels of assembly and release were observed for SIV with truncated Env in monkey PBMC, human HUT78, CEMx174, 293T, growth-arrested CEMx174 cells and macrophages resulting in production of infectious particles. We previously observed that SIVmac239T Env with a truncated cytoplasmic tail exhibited the ability to self-associate on the cell surface and assemble into a more closely packed array than full-length Env [9]. Our results indicated that the long cytoplasmic tail of the Env glycoprotein is required for incorporation of Env into lipid rafts, but Env truncation allows SIV to replicate under conditions that are non-permissive for SIV with the full length Env glycoprotein. Since SIV viruses with truncated Env glycoproteins are able to establish productive infection, lipid raft association is apparently not required for virus replication and truncated Env is assembled into infectious SIV virions even though it was not incorporated into lipid rafts. Truncation of the cytoplasmic domain of the SIV Env glycoprotein alters the conformation of the external domain and results in more stable oligomers of TM glycoprotein [45], and the truncated Env glycoprotein is more fusogenic than the full length Env [10,11]. These features for incoming virus particles may result in less dependence on the lipid composition of the viral membrane. However, a recent study reported that cholesterol-depleted HIV-1 virions exhibited a defect in internalization [46]. Taken together, the results suggest that SIV with a truncated cytoplasmic tail can overcome a restriction in post-nuclear replication events, but exhibits a defect in early replication events in human and monkey cells.

**Figure 7:**
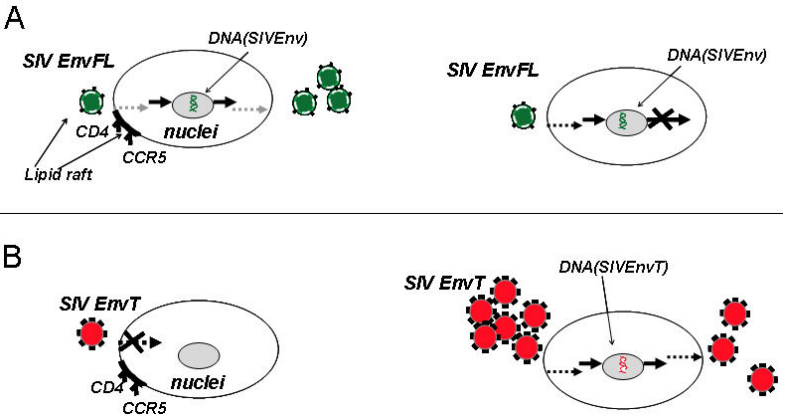
**Schematic comparison of SIV with full length and truncated Env**. Replication of SIV with full length Env (A) or with truncated Env (B) in permissive (monkey PBMC) cells (left diagrams) and non-permissive cells (brain cells, macrophages) (right diagrams). Schematic depiction of the trafficking of SIV in cells: Gray dashed arrows depicted raft-associated pathway; black dashed arrow depicts alternative pathway; black arrows depicted sites of transcription. SIV with truncated Env can overcome a restriction in an early replication step by high multiplicity of infection and productively infect cells.

Circulation of SIV with truncated Env among disease resistant primates in Africa or disease sensitive primates in Asia may indicate that this form of virus appeared when virus is adapting to new cells such as such as epithelial on brain cells, macrophages [47] or in response to factors controlling pathogenicity of virus [43]. However, experimental infection of monkeys by SIV with truncated Env showed a restricted circulation of this virus in PBMC [15,48]. Our results suggest that the restricted circulation of Env-truncated variants *in vivo *may be related to a defect in a post-entry step (Fig. 7A). The virus with full length Env has higher specific infectivity than virus with truncated Env, and is capable to establish productive infection in permissive T cells and persistent infection in non-permissive cells such as epithelial and dendritic cells or macrophages [49,50] because early steps in replication appear to be more efficient in viruses having a long cytoplasmic tail incorporated into lipid rafts domains of incoming particles (Fig. 7A, B). However, SIV with truncated Env can overcome this early restriction by high multiplicity of infection (Fig. 7B). A high multiplicity of infection would be difficult to obtain by virus with truncated Env *in vivo*, because of its sensitivity to the humoral immune response [47]. This is a possible reason why a most viruses with truncated Env were derived from tissues of immunocompromised macaques, or from brain tissue, an immune-privileged site. We suggest that the long cytoplasmic tail of the Env glycoprotein may interact with viral (p17) [1] or cellular proteins [32]. It was shown that the HIV-1 envelope glycoprotein with a long cytoplasmic tail directly influences the site of release of Gag particles in polarized epithelial cells [5] and microtubules may play an important role in assembly and maintenance of the polarized viral budding platform. Treatment of infected T cells with inhibitors of actin or tubulin remodeling disrupted Gag and Env compartmentalization within the polarized raft-like domains [51]. Co-localization of the reverse transcription complex with actin microfilaments and viral matrix was also observed during early steps in replication [28,52]. We suggest that the long cytoplasmic tail of the Env glycoprotein may affect interaction of viral core proteins with the cytoskeleton, which is important for viral relocation to the transcriptional site. Finally, our results may help to develop a strategy against pathogenic forms of HIV which could prevent the initial infection process. One example is development of topical microbicides targeted to post entry inhibition of HIV infection by interfering with Env function in an early step of virus replication [53].

## Conclusion

The present results indicate that a possible basis for defective replication of SIV with truncated Env in primates may be a restriction during an early step of replication, whereas defective replication of SIV with full length Env in human T cells may result from a restriction during a late step of replication and assembly. Comparable host-cell transcriptional responses in rhesus monkey PBMC to both types of virus infection also indicates that cells respond similarly to replication of SIV with full length or truncated Env. A mutation in the Env sequence relating to T cell adaptation alters SIV properties including sensitivity to neutralization, level of Env incorporation, rate of replication and association with lipid rafts during the course of adaptation to human cells.

## Methods

### Cell and virus stocks

The recombinant monkey cell lines sMAGI and human MAGI-R5 were obtained from the NIH AIDS Research and Reference Reagent Program. T-cell line HUT78 and T-B hybrid cell line CEMx174 were obtained from the American Type Culture Collection (Manassas, VA). The recombinant epithelial human cell line JC53-BL (indicator cell line), which is a derivative of HeLa cells that expresses high levels of CD4 and coreceptors CCR5 and CXCR4 [54], was obtained from Dr. J. Kappes (University of Alabama, Birmingham). The human 239T cell line was kindly provided by Dr. S. L. Lydy. Rhesus monkey PBMCs were separated by centrifugation of whole blood over LSM Lymphocyte Separation Medium (ICN Biomedicals Inc., Costa Mesa, CA). Cells were then stimulated with concanavalin A (Con A, 5 μg/ml in RPMI 1640 containing 10% heat-inactivated fetal calf serum; interleukin-2, human (hIL-2), 10 U/ml; 10 mM HEPES; and antibiotics) for three days before virus infection. To prepare monkey macrophages, PBMC were isolated as described above. Cells (3 × 10^7 ^in RPMI 1640 containing 15% human AB^+ ^serum, 1.5 ng/ml of M-CSF, and 0.08 ng/ml of GM-CSF) were seeded into 100-mm plates or split into 24-well plate and incubated for 4 days to allow adherence of monocytes. After removal of nonadherent cells, cells were incubated for another 3–4 days before infection.

SMAGI, MAGI-R5, JC53-BL, and 239T cells were maintained in Dulbecco's minimal essential medium (DMEM) supplemented with 10% fetal calf serum and antibiotics. HUT78 and CEMx174 cells were maintained in RPMI 1640 supplemented with 10% fetal calf serum and antibiotics, and buffered by 10 mM HEPES.

Preparation of cloned SIV stocks, standardization of virus titers, and conditions for virus infection were done as described earlier [25]. It is commonly accepted to use the infectious titer [55] or TCID50 [56] for measurement of the quantity of SIV and HIV. However, these methods are not able to precisely compare viruses with different properties such as rate of replication or production of non-infectious particles. We used infectious the index (IU/ng) which is the ratio between infectious titer and core antigen, which is taking both of these characteristics into consideration.

Prior to cell infection, virus preparations were treated with 200 U/ml RNase-free DNase I in growth medium containing 10 mM MgCl_2 _for 30 min 37°C to remove contaminating proviral DNA [57]. Plasmid pHIVSG3 containing the HIV-1 provirus (SG3) with a deleted env gene was a generous gift from Beatrice Hahn. Plasmid pCMV-GP encoding the Ebola envelope protein GP was provided by C. Yang. The plasmid pRB239ser-787 which carried a mutation in the long cytoplasmic tail of the Env glycoprotein of SIVmac239 C787S to eliminate palmitylation (see below) was digested by *Nhe*I and *Bgl*II and the resulting fragment with the mutation was introduced in plasmid p3'239 which contained the 3' portion of molecularly cloned SIVmac239 [25] in identical restriction sites. The plasmid, designated p3'239ser-787, was used to obtain a mutant virus as described above.

### Construction of recombinant vaccinia viruses

Recombinant vaccinia viruses expressing the SIVmac239-Env or SIVmac239-EnvT were described previously [9]. For the construction of SIVmac239-EnvC787S the codon TGC (cysteine) was changed to AGT (serine) by overlapping PCR. The env gene was amplified from p239SpE3' (NIH AIDS Research and Reference Reagent Program) by using the following primers: primer A (with *EcoR*I restriction site), CAAAGAATTCAGTATGGGATG; primer B (overlapping primer), GGTTTCTACTGTTGCTGA; primer C (overlapping primer), TCAGCAACAGTAGAACC; and primer D (with restriction site of *Stu*I), GTATTTCTAGGCCTCACAAGAG. Primers B and C carried the codon to be changed. Two PCR amplifications were carried out by using the p239SpE3' plasmid as template. Each PCR was carried out for 25 cycles with steps of 1 min at 95°C, 2 min at 50°C, and 3 min 72°C. The PCR products were purified with a gel extraction kit (Qiagen) according to the manufacturer's protocol. The two overlapping PCR fragments AB and CD were joined by mixing and a PCR reaction with the external primers A and D was performed. The resulting PCR fragment AD was initially cloned in the pDrive vector (Qiagen). The plasmid was digested with *EcoR*I and *Stu*I and the fragment was cloned in vector pRB21. The resulting plasmid was designated pRB239ser-787 and used for preparation of recombinant vaccinia virus as described [58].

### SIV infection

Conditions for infection with SIV were described previously [25]. At 24 h before infection, 3 × 10^6 ^cells were treated with 5 μg/ml aphidicolin, and cells were inoculated with SIV for 2 h in medium with 15 ug/ml DEAE-dextran with or without aphidicolin.

After this incubation unbound virus was removed by three washes and medium with or without aphidicolin was added. For 3 day samples, new medium with 5 μM AZT and with or without aphidicolin was added after 1 day. After 1 and 3 days, the culture supernatant and cells were harvested from each well and used for assays. The p27 content was determined by ELISA Core Antigen assay (Coulter Corporation). The infectivity of virus particles was determined by β-galactosidase assays in JC53-BL [54], MAGI-R5 or SMAGI cells [29,59].

### Supernatants, cell and nuclear extracts

The supernatants were harvested and clarified by centrifugation at 3.5 k for 20 min (GS-15R, Beckman). Cells were washed three times with PBS and lysed in RIP buffer [9] and production of Gag antigen was analyzed by SIV Core Antigen Assay (Coulter Corporation). The culture supernatants were also assayed for RT activity by colorimetric reverse transcriptase assay (Roche).

To prepare cell extracts, the cells (3 × 10^6^) were suspended in 0.01 M NaCl, 0.01 M MgCl_2 _[pH 7.4] for 10 min on ice and then lysed by addition of NP-40 to 1% followed by vortexing as described previously by [36]. Nuclei were recovered by centrifugation at 12,000 × g for 2 min, and nuclear DNA was extracted with a Dneasy Tissue kit (Qiagen) and analyzed by RT-PCR.

### RNA preparation and microarray analysis

Total RNA was extracted from cells by using the Rneasy kit (Qiagen, Valencia, CA), according to the manufacturer's protocol. Reverse transcription, second-strand synthesis, and probe generation were accomplished by standard Affymetrix protocols. The Gene Chip HG_U133_Plus 2.0 array (Affymetrix), containing ≈ 33,000 known genes, was hybridized, washed, and scanned according to Affymetrix protocols within the Baylor Affymetrix Core facility. Changes in cellular mRNA levels after SIV infection were compared with mRNA levels in controls that were identically plated, treated, and incubated in the absence of virus. GeneSpring, version 6.2 was used to normalize and scale results and compare viral responses to those of controls. The program clusters increases or decreases of expression levels as the fold change relative to control.

### Real-time PCR amplification for SIV

Quantification of proviral DNA from infected cells was performed by real-time PCR using the TaqMan amplification system as described elsewhere [37]. For PCR amplification for the SIV gag region, forward and reverse PCR primers were SIVgagF AGTACGGCTGAGTGAAGGCAGTA and SIVgagR GACCCGCGCCTTTATAGGA, respectively. The fluorogenic SIVgag probe CGGCAGGAACCAACCACGACG was modified with FAM/TAMRA [37]. PCR amplification for the SIV 2LTR region was carried out. Forward and reverse PCR primers were U3U5-2LTRF GGAACGCCCACTTTCTTGATGTATA and U3U5-2LTRR CGGCGGCTAGGAGAGATG. The fluorogenic 2LTR probe was SIV U3U5-2LTRM2 FAM AACACACACTAGCTAATACAG. Nuclear DNA samples corresponding to equal numbers of cells infected by SIV were analyzed in parallel. Fluorescence was recorded as a function of PCR amplification cycle. Quantitative SIV determinations were made by comparison with a standard curve produced by using serial dilution of plasmid DNA with a 1890 bp region of the SIVmac239 gag gene [60].

### RNA isolation and cDNA synthesis

To determine the mRNA transcription profile of selected genes the relative quantitative real-time PCR was performed. PBMC were harvested from cell culture and lysed immediately with 350 μl of lysis buffer from the MagNA Pure LC RNA Isolation Kit III (Tissue) (Roche), then frozen at -80°C. Collected samples were extracted with a MagNa Pure LC – robotic workstation (Roche Molecular Biochemicals) with the same kit using the external lysis protocol. Total RNA was eluted in 60 μl of water and optical density measurements were taken immediately. All total RNA was reverse-transcribed using a High-Capacity cDNA Archive Kit Protocol (Applied Biosystems Inc.).

### Quantitative Real-Time PCR Analysis

The reaction was carried out on a 384-well optical plate (Applied Biosystems) in a 20-μl reaction volume containing 30 ng of cDNA per reaction with TaqMan Universal PCR Mastermix, Applied Biosystems. All sequences were amplified using the Applied Biosystems 7900HT Sequence Detection System with the PCR profile: 50° for 2 min, 95°C for 10 min, followed by 45 cycles at 95°C for 15 s, and 60°C for 1 min. Samples were tested in duplicate, in parallel with the housekeeping gene GUSB. For relative quantitation delta-delta Ct analysis was applied to recalculate the fold differences between samples.

### Primer and probe sequences

Oligonucleotide primers and probes for IL-2, IL-4, IL-6, TNF-α, IFN-α, TNF-β, and Mx were used as described by [61]. For IL-10 were used two sets of primers and probes. For IL-10 assay a first set of oligonucleotide primers and probe were used as described by [62] and a second set described below. For other assays oligonucleotide primers and probes were designed using the Primer Express Software (Applied Biosystems) based on published rhesus macaque sequences.

IFNγ : F – GAAAAGCTGACCAATTATTCGGTAA,

R – GCGACAGTTCAGCCATCACTT,

P – 5'FAM – CCAACGCAAAGCAGTACATGAACTCATCC – TAMRA-3';

IL10: F – GTCATCGATTTCTTCCCTGTGAA

R – CTTGGAGCTTACTAAAGGCATTCTTC

P – 5'FAM – CCTGCTCCACGGCCTTGCTCTTG – 3'TAMRA;

IL-12p40: F – TGAAGAAAGACGTTTATGTTGTAGAATTG,

R – TGGTCCAAGGTCCAGGTGAT,

P – 6FAM – CTGGTACCCGGATGC – MGBNFQ

IL-7: F – GATGGCAAACAATATGAGAGTGTTCT,

R – CAATTTCTTTCATGCTGTCCAATAAT,

P – 6FAM – TGGTCAGCATCGATC-MGBNFQ;

IL-15 F – AGCTGGCATTCATGTCTTCATTT,

R – CACCCAGTTGGCTTCTGTTTTAG,

P – 6FAM – CTGTTTCAGTGCAGGGC – MGBNFQ.

Primers and probes were obtained from Applied Biosystems with assay ID as follows: SPIB – Hs00162150_m1; PU.1 – Hs00231368_m1; IRF1 – Hs00971959_m1; IRF2 – Hs00180006_m1; IRF3 – Hs00155574_m1; IRF4 – Hs00277069_m1; IRF5 – Hs00158114_m1; IRF7 – Hs00185375_m1; IFNA1 – Hs00256882_s1. All these assays were designed based on human sequences, and before implementation all assays were validated for rhesus macaques.

### Isolation of lipid raft proteins

Radioactively labeled cells expressing different recombinant Env glycoproteins were washed three times with ice cold PBS+++ and then lysed on ice in 750 μl TNE buffer (10 mM Tris HCl pH 7.5, 150 mM NaCl, 5 mM EDTA with a protease inhibitor cocktail (Roche)) with 0.5% (v/v) TX-100 for 20 min as described previously [63]. The lysate was passed 10 times through a 25 G needle on ice and subsequently centrifuged at 8000 × g for 10 min at 4°C. The supernatant was brought to 40% sucrose by adding 750 μl of 80% (w/v) sucrose in TNE, loaded into the bottom of a SW41 centrifuge tube and overlaid with 6 ml of 30% (w/v) sucrose in TNE and 3.5 ml 5% (w/v) sucrose in TNE. The samples were spun to equilibrium at 200,000 × g for 13–16 hr. Eleven fractions with volume of each 1 ml were collected started from the top of the gradient and subjected to immunoprecipitation with monkey anti-SIV serum. Samples were analyzed with an SDS-8% PAGE gel and subsequent autoradiography.

### MTT assay

For the MTT assay, aphidicolin treated or untreated CEMx174 cells in 96-well plates were infected with SIV. After 24 h or 72 h incubation, 10 μl of MTT (10 mg/ml) reagent was added to 100 μl of medium in each well. After 4 h incubation at 37°C, 100 μl acidic isopropanol (0.04 M HCl in absolute isopropanol) was added. The absorbance was read in a computer-controlled photometer. The absorbance at 690 nm was automatically subtracted from the absorbance at 540 nm to eliminate the effects of non-specific absorption. The MTT assay, which provides an indication of mitochondrial integrity and activity, is not dependent on the cell cycle.

## Competing interests

The author(s) declare that they have no competing interests.

## Authors' contributions

Author ANV designed experiments, carried out most the experiments and wrote the manuscript. Author AW carried out most experiments for the lipid raft section and drafted this section. NLK assisted in RT-PCR experiments. VK and BY carried out microarray analysis. RWC suggested experiments and revised the manuscript.
